# Assessing the Effects of Agronomic Management Practices on Soybean (*Glycine max* L.) Post-Grain Harvest Residue Quality in the Lower Mississippi Delta

**DOI:** 10.3390/plants10071337

**Published:** 2021-06-30

**Authors:** Srinivasa R. Pinnamaneni, Saseendran S. Anapalli

**Affiliations:** USDA-ARS, Sustainable Water Management Research Unit, Stoneville, MS 38776, USA; SASEENDRAN.ANAPALLI@USDA.GOV

**Keywords:** soybean, residue composition, protein, lignin, detergent fiber, irrigation, planting pattern, relative feed value

## Abstract

Livestock producers often resort to either baling or grazing of crop residues due to high hay prices and reduced supply of other forages and silage in the markets. Soil-water-crop management practices can affect residue nutrient qualities for its use as cattle feedstock. A two-year study (2018–2019) was conducted to investigate the effects of irrigation (AI, all row-irrigation; ARI, alternate row irrigation; and RF, rainfed) and planting pattern, PP (SR, single row; and TR, twin-row) on soybean (maturity group IV cv. 31RY45 Dyna-Gro) post-grain harvest residue quality such as crude protein (CP), acid detergent fiber (ADF), neutral detergent fiber (NDF), acid detergent lignin (ADL), net energy for maintenance (NEM), net energy for gain (NEG), net energy for lactation (NEL), total digestible nutrients (TDN), and relative feed value (RFV). Irrigation has a significant effect on CP, ADF, NDF, and TDN while PP affected only NDF. All the above parameters were significantly affected except NEM by the contrasting climate conditions, particularly during July through August coinciding with early crop reproductive stages and maturity. The RFV values ranged from 70.4 to 81.6 and this lower range was attributable to nutrient translocation to seeds and higher lignification during plant senescence towards the grain filling stage of the crop as good quality hay records over 120 RFV. These results indicate that both irrigation and weather during soybean seed development can alter post-grain harvest residue quality parameters, thereby playing critical roles in its RFV.

## 1. Introduction

Soybean [*Glycine max* (L.) Merr.] is an important short-duration grain and forage crop for human consumption and livestock feeding in the Leguminosae family. It has a long history of use as a primary forage crop in the United States, owing to its high-quality proteinaceous hay [1]. With the advent of perennial forage crops, use of annual soybean has become less popular. Since the 1960s, soybean has been predominantly grown as a food crop for its high-value protein and oil contents. Although baling of soybean residue is not a common practice, when higher hay prices are prevalent and corn residue is limiting, producers harvest soybean residue to get additional returns [2]. 

A multi-year study conducted in the Midwest USA demonstrated that removal of corn residue by baling or grazing will not affect soil fertility loss or yield returns of the following crop [3]. Like corn, the post-grain harvest residue of soybean also could be an excellent cattle-feed alternative through the summer months if handled appropriately and may accrue additional income for the farmers. In this direction, it was reported, some livestock producers mix the shredded soybean residue with distiller’s grain for feeding livestock or use it as roughage [2]. Some livestock producers in the North-central and North-eastern USA were reported ensiling soybean due to the high cost of perennial hay such as alfalfa and red clover [4].

A study conducted in Ontario, Canada, demonstrated that yearly about 1.1 million tons of corn, soybean, and winter wheat residue could be sustainably harvested without affecting the soil organic matter content [5]. Moderate to low grazing on soybean did not influence either soil carbon or organic matter content. It has been reported that harvesting/baling soybean residue will not impact nutrient loss, soil erosion, soil quality, water loss, and yield if the threshold level of 5 tons of residue per ha is maintained [6]. It has been estimated that for every ton of soybean grain, about 1.22 tons of residue is produced [2,6]. Hence, based on the soybean production levels of 2019, about 125 million tons of soybean residue is potentially available in USA for grazing or baling. Several studies in the past evaluated soybean biomass at different growth stages for feedstock quality and quantity [7,8,9,10] and found R6-R7 stage harvested biomass is superior in quality [7,10]. The crude protein (CP) content of soybean hay varied with its source-tissue: ranged from 12 to 14% for stems, 19 to 20% for leaves, and 12 to 27% for pods, based on the phenological harvest stage [11]. Studies examining nutritional qualities of soybean post-harvest crop residue as a potential cattle-feed alternative are lacking in the literature.

Soybean growers in Mississippi and adjoining states in the Mid-Southern USA plant in either single row (SR) or twin-rows (TR) [12,13]. Soybean seeds are planted on a single row on ridges spaced by about 100 cm in the SR system, and in the TR, the single rows were replaced by twin rows separated by about 25 cm. Managing both planting pattern (PP) and irrigation are two critical crop management practices that can optimize seed yield in the Midsouth USA [12,13]. However, possible effects of such changes in management on soybean residue quality is not available. Munoz et al. (1983) reported an association of pre-grain harvest biomass yield with planting density, but inverse relation to its digestibility in cattle. The same study also revealed a decline in digestibility with the delays in harvesting. It was reported that the leaf fraction in the harvested forage-biomass reduced from 70.8% at the R1 stage (beginning bloom) to 16.8% at the R7 stage (beginning seed maturity) [14]. However, the stem portion increased from 29.2% at R1 to 38.3% at the R5 stage (beginning seed) and then reduced to 28.3% at R7 stage, (beginning maturity). It may be appropriate to harvest forage soybean no later than the R7 stage [10,15]. 

Although a few reports are available on the effects of irrigation and water stress, as above on soybean biomass composition, there has been no research on the combined effects of irrigation and PP on post-grain harvest residue quality parameters. We hypothesized that the PP combined with different irrigation levels will subject the crop to a unique micro-environment due to early canopy closure enhancing light interception, carbon assimilation, and nutrient uptake that can impact biomass quality parameters such as neutral detergent fiber (NDF), acid detergent fiber (ADF), acid detergent lignin (ADL), net energy for lactation (NEL), total digestible nutrients (TDN), and relative feed value (RFV). Therefore, the objective of the current study was to assess the effects of SR and TR plantings with three levels of irrigation (AI, all row-irrigation; ARI, alternate row irrigation; and RF, rainfed) on soybean post-grain harvest residue composition for its suitability as forage in the Lower Mississippi Delta region. 

## 2. Results

### 2.1. Weather Across Crop Seasons

**Figure 1:**
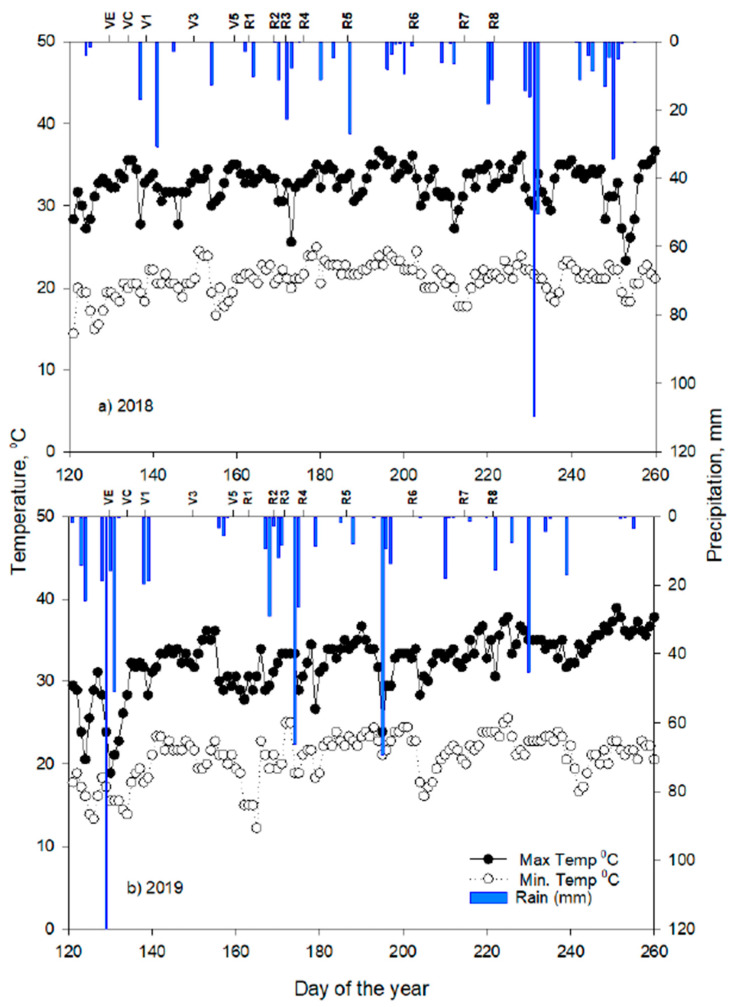
Measured air temperature (left *y* axis) precipitation (right *y* axis) and phenological stages (top *X*-axis) in (**a**) 2018 and (**b**) 2019 soybean crop seasons at Stoneville, MS, USA.

**Figure 2:**
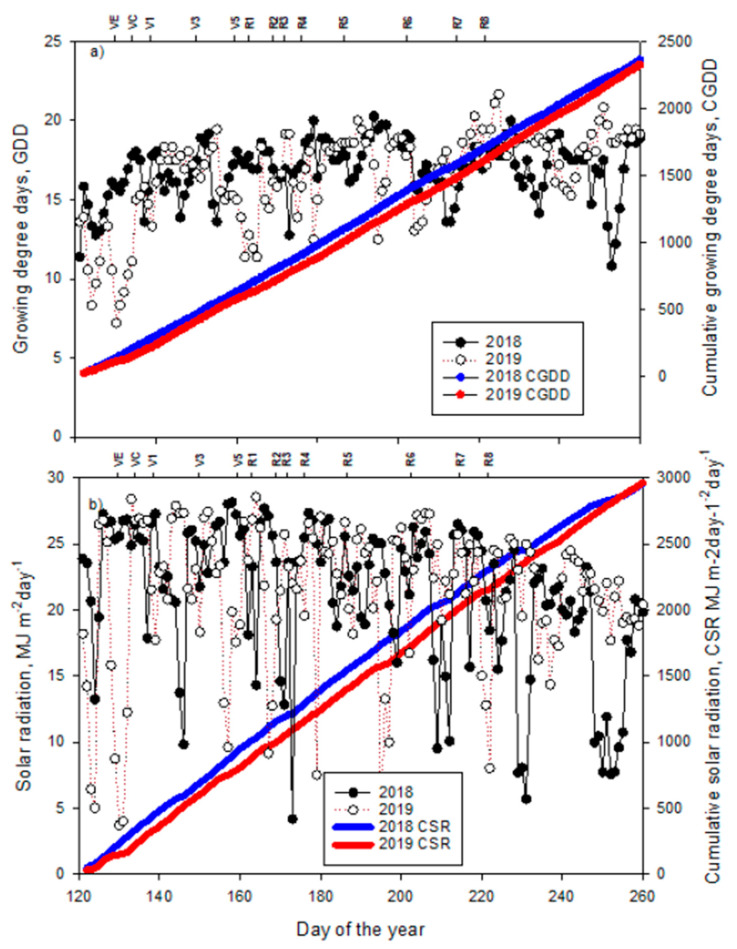
Measured (**a**) growing degree days (GDD, °C) computed using 10 °C as base temperature, cumulative growing degree days (CGDD) and (**b**) monthly averaged daily solar radiation, cumulative solar radiation (CSR) in 2018 and 2019 soybean growing seasons at Stoneville, MS, USA.

The observed weather during the two cropping seasons (May–September in 2018 and 2019) differed significantly (Figure 1 and Figure 2). A precipitation of 147 mm during June–July, 2018 compared to 273 mm during the same period in 2019, which coincided with beginning bloom (R1) and full pod (R4) stages. Conversely, the month of August in 2018 season had 2.5 times higher precipitation than that of 2019 (231 mm in 2018 vs. 92 mm in 2019), and it coincided with the R4 -R8 stages. On an average, about 1.5 °C higher mean minimum and maximum air temperatures were recorded in 2018 vis a vis 2019 during the months of June and July, which coincides with the peak flowering period. The 2018 peak flowering period recorded 51 additional growing degree days (GDD, °C) than in 2019 (782 in 2019 vs. 833 in 2018). In the case of solar radiation, 37% lower radiation was received in the 2018 season compared to that of the 2019 season during the flowering phase (2018: 15.8 MJ m^−2^ day^−1^ vs. 2019: 21.6 MJ m^−2^ day^−1^). The differences in weather during the two crop seasons, in particular, the flowering and maturity phases, were reflected in the ANOVA tests for most of the forage quality traits (Table 1).

### 2.2. Analysis of Variance (ANOVA) for Forage Quality Traits

The ANOVA (Table 1) showed that irrigation, PP, crop season, and their interactions had significant effects on some forage quality components but not on others. For instance, irrigation levels had affected biomass, dry matter, crude protein, ADF, and NEL. However, PP had affected NDF and NEL. The most significant effect was observed for the crop season on all the traits studied except for NEM. The degree of variation among the many forage quality traits is probably attributable to the high variation in precipitation, temperature, solar radiation, and GDD among the two crop seasons coinciding with the flowering and pod development and leaf senescence (Figure 1 and Figure 2). The interactions were mostly non-significant except for irrigation and PP affecting ADF and NDF, while the interaction effect of irrigation and crop season was significant for biomass yield, dry matter, crude protein, and ADF.

### 2.3. Biomass Yield and Forage Quality as Influenced by Irrigation

Irrigation and TR had a favorable effect on seed yield in both the crop seasons, as reported by Pinnamaneni et al. [12] where, in the grain yields, were: 4.8 Mg ha^−1^ in AI-TR, 4.7 in Mg ha^−1^ in ARI-TR, 4.2 Mg ha^−1^ in AI-SR, 4.1 Mg ha^−1^ each in RF-TR and ARI-SR, and 3.6 Mg ha^−1^ in RF-SR. The average biomass yields among the irrigation and PP treatments were AI: 5.3 Mg ha^−1^ in TR vs. 5.2 Mg ha^−1^ in SR, ARI: 5.1 Mg ha^−1^ in TR vs. 5.0 Mg ha^−1^ in SR, and RF: 4.5 Mg ha^−1^ in TR vs. 4.6 Mg ha^−1^ in SR. The CP level among the irrigated treatments ranged between 7.8%–7.9% in 2018, but they were significantly lower in rainfed treatment that ranged between 7.5%–7.6%. In the 2019 crop season, crude protein level was higher in AI and ARI treatments ranging between 8% and 8.2%, while in RF, it varied between 7.7 and 7.8% (Table 2). The NDF had an inverse relationship with irrigation, and significantly higher levels were observed in the RF-TR and RF-SR treatments with values of 66.7% and 65.2% in 2018 and 63.5% and 64.2% in 2019. Similar trends were observed for ADF content in both the crop seasons of the study, where the RF treatments recorded 4.8% higher in 2018 and 6.2% higher levels in the 2019 season (Table 2). Irrigation levels did not affect lignin levels. However, it ranged between 13.2%–14.5% in 2018 while it was between 10.6% and 11.6% in 2019. No significant differences were observed for NFC content between the irrigated and rainfed treatments in both the crop seasons. The TDN is a key quality trait and is positively affected by irrigation, although significant crop season-wise differences cannot be ignored as most of the treatments had significantly higher TDN in the 2019 season than that of 2018, probably due to higher precipitation and lower mean temperatures during pod development (Table 3). In 2018, the irrigated treatments recorded 5% higher over rainfed, while 6.9% higher TDN levels in irrigated treatments were observed. The mean TDN values were 46.3% in AI-TR vs. 46.6% in AI-SR, 44.9% in ARI-TR vs. 45.9% in ARI-SR, while RF-SR had 42.9%, which was the lowest TDN recorded. The NEL was significantly higher in irrigated treatments than RF in both the crop seasons by about 6.4%. The mean NEL values were 0.32% in AI-TR vs. 0.33% in AI-SR, 0.30% in ARI-TR vs. 0.32% in ARI-SR, while RF-SR had 0.31%, and the lowest TDN was recorded in RF-TR at 0.29%. In NEM and NEG, the effect of irrigation was negligible. The season-wise differences for all the quality traits were significant except for NEM and ash content, probably due to the differences in measured climate (variability in precipitation and solar radiation) during flowering and post-flowering stages in 2018 and 2019. Statistically significant interactions of irrigations with crop seasons were observed for biomass yield, dry matter, and NDF.

### 2.4. Biomass Yield and Forage Quality as Influenced by Planting Pattern (PP)

In an earlier report from this study, Pinnamaneni et al. [12] communicated that averaged across two seasons and three irrigation regimes, TR enhanced grain yields by 13% over SR (4.5 Mg ha^−1^ vs. 4.0 Mg ha^−1^) due to better plant stand establishment and interception of photosynthetically active radiation. However, in the case of biomass production per unit area, the results were different, which is not unexpected. In the case of biomass production, the TR recorded significantly higher biomass by 5.7% in 2018 (3.7 Mg ha^−1^ vs. 3.5 Mg ha^−1^) and 1.7% higher in 2019 seasons, which is statistically insignificant (4.1 Mg ha^−1^ vs. 4.0 Mg ha^−1^). Most of the forage quality traits were not impacted by PP, except for NDF and NEL (Table 4). The TR PP had a significant positive impact on NDF as TR treatments had 3.1% and 2.1% higher values in 2018 and 2019, respectively (Table 2). However, the NEL, an estimate of the energy value of a feed used for maintenance and milk production after digestive and metabolic losses, was higher in SR PP than that of TR by 3.6% in 2018 and 3.9% in 2019 (Table 3).

### 2.5. Relative Feed Value (RFV) as Influenced by Irrigation and PP

The RFV is widely used in assessing the energy content of hay while deciding the grades in hay markets. The increase of fiber components (NDF and ADF) and sharp decline in CP after R6 stage is contributing to lower hay quality grades. The differences among the irrigation and PP treatments were non-significant; however, the RFV was significantly different across the two crop seasons. Significantly higher RFV was recorded in 2019 (2018: 73–78.9%; 2019: 78.2–84.6%) (Table 3).

## 3. Discussion

The biomass/residue yield after grain harvest was positively affected by irrigation in both the crop seasons (5.7% increase in irrigated plots over RF plots in 2018 and 1.7% higher in 2019). However, there is no impact of PP on biomass yield. Hintz et al. (1992) reported greater biomass yield coupled with lower CP content and inconsistent effects on fiber parameters (NDF and ADF) with a narrow row spacing of 20 cm. In our study, the effect of irrigation and PP on some of the forage quality parameters like CP, NDF, TDN was significant. However, the crop season-wise differences for almost all the forage quality parameters were highly significant due to contrasting patterns of precipitation in 2018 and 2019, coupled with temperature differences during post-flowering growth stages. Air temperature, moisture deficit, solar radiation, and soil nutrient status in the plant environment have a profound influence on soybean biomass quality by varying leaf/stem ratios, and changes in composition constituents of the tissues [16,17]. The soluble sugars start accumulating when lower than optimum temperature exist because of their lower sensitivity in photosynthesis than in expansion-growth, so that the photosynthate unused in expansion growth will be stored in leaf-cell vacuoles. On the other hand, a temperature rise generally increases the rate of plant development and reduces leaf/stem ratios and digestibility. It was reported that a 1 °C increase in temperature will reduce digestibility by 3–7 g kg^−1^ with no significant effect on CP levels [18,19]. The steep decline in digestibility linked with higher temperatures is generally attributed to higher NDF levels as vindicated in this study as higher temperatures of about 1.5 °C in crop season in 2018 lead to 3.5% higher NDF and 21% higher lignin content. It is obvious that the forages grown under more than optimum temperatures are usually less digestible owing to higher levels of lignin [16,20]. 

It was recommended to harvest forage soybean between R6- full seed and R7-beginning maturity stages to get the advantage of high biomass and CP [4,17]. Whole-plant crude protein concentration of grain soybean can increase significantly beyond R5-beginning seed stage as protein becomes concentrated in the pods while CP in other tissues such as stems, and leaves remains constant or decreases only slightly [15]. Both protein nitrogen and soluble carbohydrates are translocated out of leaves as they age. Moisture deficit stress, based on the severity and timing, typically slows maturation in alfalfa [17]. The effects of moisture deficit stress on CP levels have been inconsistent. Under prolonged moisture stress, leaf senescence hastens up, leading to nutrient translocation from leaves to roots, thus resulting in low forage quality.

In this study, the crop was harvested at R8 (full maturity), hence the higher lignification of the stem and significant leaf fall due to senescence (near 100%) at the time of harvest has probably contributed to lower levels of CP (7.5–8.2%). It is known that harvesting soybean during early reproductive development (R1 to R5 stages) may result in low dry matter yields [15,16]. Soybean biomass harvested at beginning grain maturity (R7) is comparable in CP, NDF, ADF, and ADL to alfalfa hay harvested at an early flowering [21] and has potential as a high-quality alternative forage. But the post-grain harvest residue collected in this study at R8 stage was significantly lower in quality.

A study conducted in Texas suggested that for lactating dairy cattle, a hay possessing 14% or more CP along with a RFV greater than 150 would be more appropriate [22]. By this standard, the soybean post-grain harvest residue produced in this study will miss the above criteria as both CP (7.5–8.2%) and RFV (74–85) were significantly lower due to (i) complete senescence of foliage, probably a characteristic of this grain soybean cultivar; (ii) translocation of proteins to maturing grain; and (iii) lignification of the stem progresses fast during late maturity stages of pod development. Additionally, total digestible nutrients (TDN) for the forage soybean grown in this study ranged from 42.1% to 48.2%. These results are comparable with the findings of previous studies [4,10,15]. Hence, the residue of soybean after grain harvest can be fed to the livestock directly or can be mixed with distiller’s grain so as to improve the quality. Alternatively, it can be used as a good roughage. This practice, coupled with seed sales, can significantly impact the on-farm profitability, particularly when there is shortage of hay and corn residue as well as high hay prices. Livestock producers can sustainably harvest the soybean residues while adhering to the guidelines of the local county specific soil and water conservation programs. Further, the bulk density of baled residue will be low, hence the residue value chain economics would be positive if the distance between residue production and place of consumption, i.e., dairies is less to avoid high transportation costs. It is anticipated that the crop residues are expected to play a greater role once viable biomass conversion technologies to advanced biofuels are in place [23]. 

## 4. Materials and Methods

### 4.1. Field Conditions and Crop Management

Field experiments were conducted in 2018–2019 at the USDA-ARS experiment farm located in Stoneville, Mississippi, USA (33° 42′ N, 90° 55′ W, elevation: 32 m above mean sea level) in a Dundee silt loam (21.54% sand, 57.62% silt, and 21.04% clay; fine silty, mixed, active, thermic Typic Endoaqualfs) soil. The physical and chemical parameters of the top 30 cm soil of the experimental field are given in Table 1. The bulk density of the soil was 1.36 g cm^−3^ and field-saturated hydraulic conductivity (K_fs_) ranged between 0.36 and 1.49 cm hr^−1^ (Saturo Infiltrometer, Meter Group Inc, WA, USA). Soybean maturity group IV cultivar 31RY45 Dyna-Gro was planted in a split-plot arrangement in a randomized complete block design with six replicates. The main plots were three irrigation regimes (i) AI, (ii) ARI, and (iii) RF, while the subplots consisted of two planting patterns (PP): (i) SR, seeds planted on seedbeds in single rows spaced at 102 cm apart, and (ii) TR, in which two rows spaced at 25-cm apart substituted the single-row of SR and the planters were set to achieve a plant population density of approximately 336,000 plants ha^−1^. The planting dates were 8 May 2018, and 2 May 2019, while the crop was harvested on 21 September 2018, and 27 September 2019, respectively. Plot size was 40 m × 3.9 m. Tillage and weed management was done as described in Pinnamaneni et al. [12]. In 2018, a total of 220 mm of irrigation was applied through a flow meter (Mc Propeller flowmeter, McCrometer Inc, CA USA) in the AI plots in four irrigation events of 55 mm each applied through every furrow on 15 May, 20 June, 6 July, and 3 August, while the ARI plots received about 50% of water per row on the same dates but in every other furrow, amounting to total water applied of about 115 mm against 220 mm in the FI. In 2019, total irrigation applied was 152 mm in the AI plots, in three irrigation events of 51 mm each on 10 June, 29 July, and 7 August, while in ARI plots, 75 mm of water was applied on the same dates. Irrigation was stopped at the R6 growth stage in both years. Above ground biomass along with grain was harvested at R8 stage from a 1 m^2^ section of each plot at three random locations, avoiding the row ends and border rows. The samples were dried for two weeks in a greenhouse and passed through a soybean thresher (Almaco Model: LPRUM8G, Nevada, IA, USA). The resultant residue consisting of majorly stem portion and pod shells other than the grain was used for this study. Weather data were collected from a weather station located within a radial distance of about 1.5 km from the experiment site, that is, Mid-South Agricultural Weather Service, Delta Research and Extension Center, Stoneville, Mississippi. The growing degree days (GDD) in °C were calculated using a base temperature of 10 °C [24,25]. 

### 4.2. Forage Quality Analysis

The post-grain harvest soybean residue was dried at 60 °C for 4 h in forced air ovens and ground using a Wiley Mill (Thomas Model 4 Wiley Mill, Thomas Scientific, Swedesboro, NJ, USA) with a 6-mm sieve. A subsample of the coarse ground samples was finely ground to pass a 1-mm sieve. Forage analyses were conducted by near-infrared spectroscopy (NIRS) using Foss NIR Systems Models XDS or 6500 with ISIScan v.4.6.12 (FOSS Analytical A/S, Denmark) to determine CP, ADF, NDF, ADL, neutral detergent insoluble crude protein (NDICP), starch, fat, ash, and expressed on a dry matter basis. Global NIRS calibrations (Dairy One Cooperative, Ithaca, NY, USA) were originally developed according to the principles in AOAC methods (989.03, 991.01) and transitioned to local calibrations using WinISI v.4.6.11 similar in approach to Schenk et al. (1997). The non-fiber carbohydrates (NFC) are calculated as 100% − (CP% + (NDF% − NDICP%) + Fat% +Ash%). NEL, an estimate of the energy value of a feed used for maintenance and milk production in lactating cattle is estimated from TDN [26]. Both NEM and NEG were estimated following the National Research Council report of 2001 [27]. To compare the quality between the different irrigation and PP treatments, the relative feed value (RFV), an objective measurement of forage quality that estimates digestible dry matter (DDM) from ADF, and calculates the DM intake potential (as a percent of body weight) from NDF was calculated as follows [28]:

Relative Feed Value (RFV) = (DDM × DMI)/1.29; (1)

Dry matter digestibility% (DDM) = 88.9 − (0.779 × ADF);(2)

Dry Matter Intake (DMI) = 120/NDF;(3)

where ADF and NDF are expressed as percentage of dry matter (DM).

### 4.3. Statistical Analyses

Data were subjected to ANOVA using PROC MIXED in Statistical Analysis System (SAS^®^ version 9.4; SAS Institute Inc., Cary, NC, USA). The crop season, irrigation, planting pattern were considered as fixed effects and the random effects are replication, irrigation × crop season, planting pattern × crop season, and irrigation × planting pattern × crop season. Treatment means were separated at the 5% level of significance using Fisher’s protected least significant difference (LSD) test. The interactions involving combinations of the crop season, PP, and irrigation were significant for most of the parameters studied. Hence, the results were presented separately by crop season.

## 5. Conclusions

Our understanding of the differential levels of forage quality indicators like CP, NDF, ADF, and ADL in soybean biomass with varying soil-crop-water management is limited. The current study demonstrated that irrigation could play a significant role in altering post-grain harvest soybean residue quality indicators like CP, NDF, ADL, and NEL, while the PP has a limited role except for NDF and NEL. The study also highlighted the significant impact of seasonal weather (air temperature, solar radiation, and precipitation) due to high inter and intra-seasonal variability. This result is further exacerbated since leaf senescence hastens up at beginning maturity (R7) and beyond. The RFV could be a good indicator of the forage value of soybean residue, which helps in decision making on residue harvesting and utilization.

## Figures and Tables

**Table 1:** Significance of the main effects of irrigation regimes, crop season, and planting pattern (PP) and their interactions on biomass quality traits at Stoneville, MS during the 2018–2019 crop seasons.

Source of Variance	df	Biomass	Dry Matter	Crude Protein	ADF (%)	NDF (%)	Lignin	NFC (%)	TDN (%)	NEL	NEM	NEG	Ash
Irrigation level	2	*	*	*	*	*	ns	ns	ns	*	ns	ns	ns
PP	1	ns	ns	ns	ns	*	ns	ns	ns	*	ns	ns	ns
Crop season	1	*	*	*	*	**	**	**	*	**	ns	*	ns
Irrigation level * PP	2	ns	ns	ns	ns	ns	ns	ns	ns	ns	ns	ns	ns
Irrigation level * crop season	2	*	*	ns	ns	ns	ns	ns	ns	ns	ns	ns	ns
PP * crop season	1	ns	ns	ns	ns	*	ns	ns	ns	ns	ns	ns	ns
Irrigation level * PP * crop season	2	ns	*	ns	ns	ns	ns	ns	ns	ns	ns	ns	ns

ns: non-significant; * Significantly different at *p* ≤ 0.05 level; ** Significantly different at *p* ≤ 0.01 level. ADF: Acid detergent fiber; NDF: Neutral detergent fiber; NFC: Non fiber carbohydrates; TDN: Total digestible nutrients; NEL: Net energy for lactation; NEM: Net energy for maintenance; NEG: Net energy for gain.

**Table 2:** Effect of irrigation treatments (AI, ARI, and RF) and planting pattern (SR, single-row and TR, twin-row) on soybean biomass yield and composition. PP: Planting pattern; AI is all-row irrigation, ARI is alternate row irrigation, and RF is rainfed.

Treatment	PP	Biomass Yield (Mg ha^−1^)		Dry Matter (%)		Crude Protein (%)		ADF (%)		NDF (%)		Lignin (%)		NFC (%)	
		2018	2019	2018	2019	2018	2019	2018	2019	2018	2019	2018	2019	2018	2019
AI	SR	5.0a	5.4a	94.2a	92.8a	7.9a	8.1a	49.0b	48.0b	60.9c	59.5c	13.8a	11.6a	13.1a	17.1a
	TR	5.1a	5.5a	94.3a	93.1a	7.8b	8.2a	48.5b	47.0b	64.2b	61.6b	13.4c	10.8b	13.3a	16.2a
ARI	SR	4.7b	5.2b	94.4a	93.7a	7.9a	8.2a	50.7a	48.6b	61.5c	61.1b	13.2c	11.2a	14.2a	16.3a
	TR	4.9b	5.3b	94.6a	93.6a	7.9a	8.0b	51.0a	50.3a	63.5b	62.2b	14.5a	10.9b	14.4a	15.9a
RF	SR	4.3d	4.6c	92.9a	92.5a	7.6c	7.8c	49.3b	48.3b	66.7a	63.5a	13.5bc	10.6b	13.9a	17.0a
	TR	4.4d	4.7c	93.3a	92.4a	7.5c	7.7c	50.2a	47.9b	67.2a	64.2a	13.5bc	10.8b	13.6a	16.9a

Means within each column followed by the same letter or letters are not statistically different by LSD means (*p* ≤ 0.05). ADF: Acid detergent fiber; NDF: Neutral detergent fiber; NFC: Non fiber carbohydrates.

**Table 3:** Effect of irrigation treatments (AI, all row irrigation, ARI, alternate row irrigation, and RF, rainfed) and planting pattern (SR, single-row and TR, twin-row) on soybean biomass quality traits. PP is planting pattern, AI is all-row irrigation, ARI is alternate row irrigation, and RF is rainfed.

Treatment	PP	TDN (%)		NEL (%)		NEM (%)		NEG (%)		Ash (%)		RFV	
		2018	2019	2018	2019	2018	2019	2018	2019	2018	2019	2018	2019
AI	SR	44.9a	48.2a	0.25c	0.36a	0.27a	0.31a	0.05a	0.07a	6.1a	6.4a	74.3a	80.5a
	TR	44.8a	47.8a	026b	0.35b	0.27a	0.29a	0.06a	0.05a	6.2a	5.6a	74.1a	81.6a
ARI	SR	44.1a	47.8a	0.28a	0.35b	0.28a	0.32a	0.05a	0.09a	5.9a	5.9a	74.1a	77.7b
	TR	43.7b	46.2b	0.27b	0.33c	0.26a	0.29a	0.05a	0.06a	5.8a	5.7a	72.0b	76.8b
RF	SR	42.5c	43.2d	0.29a	0.36a	0.27a	0.31a	0.04a	0.05a	4.9b	4.9b	70.4d	74.0c
	TR	42.1c	45.7c	0.26b	0.32c	0.27a	0.30a	0.05a	0.08a	5.5a	6.0a	71.0c	73.7c

Means in each column followed by the same letter or letters are not statistically different by LSD means (*p* ≤ 0.05). TDN: Total digestible nutrients; NEL: Net energy for lactation; NEM: Net energy for maintenance; NEG: Net energy for gain; RFV: Relative feed value.

**Table 4:** Selected chemical properties of research fields used for experiments in Stoneville, MS, in 2018 and 2019.

Crop Season	Soil Depth (cm)	pH	Organic Matter (%)	CEC (Meq 100 g^−1^)	Mehlich-3 Extractable Nutrients (mg Kg^−1^)						
					P	K	Ca	Mg	Zn	S	Cu
2018	0–15	6.75	1.23	9.2	32	156	1168	246	1.6	6.1	1.4
2018	15–30	6.79	1.20	13.4	19	142	1758	292	1.4	5.9	1.8
2019	0–15	6.83	1.23	8.2	27	119	1003	226	1.5	5.6	1.3
2019	15–30	6.77	1.21	12.9	17	133	1617	296	1.4	5.8	2.0

CEC: Cation exchange capacity.

## Data Availability

All data presented within is the corresponding authors’ data and is available upon request.

## References

[1] Gibson L., Benson G. (2005). Origin, History, and Uses of Soybean (Glycine max).

[2] Rees J.C.W., Mary D., Keith G., Randy P., Todd W. What Is the Value of Soybean Residue?. https://cropwatch.unl.edu/2018/what-value-soybean-residue.

[3] Ulmer K.M., Rasby R.J., Macdonald J.C., Blanco-Canqui H., Rakkar M.K., Cox J.L., Bondurant R.G., Jenkin K.H., Drewnoski M.E. (2019). Baling or grazing of corn residue does not reduce crop production in central United States. Agron. J..

[4] Seiter S., Altemose C.E., Davis M.H. (2004). Forage soybean yield and quality responses to plant density and row distance. Agron. J..

[5] Kludze H., Deen B., Weersink A., van Acker R., Janovicek K., De Laporte A., McDonald I. (2013). Estimating sustainable crop residue removal rates and costs based on soil organic matter dynamics and rotational complexity. Biomass Bioenergy.

[6] Wortmann C.S., Klein R.N., Shapiro C.A. (2012). Harvesting Crop Residues. Inst. Agric. Nat. Resour..

[7] Tubbs R.S., Gallaher R.N. (2010). Row spacing and cultivar effects on yield and forage quality of fall-grown soybean. Crop Manag..

[8] Asekova S., Han S.-I., Choi H.-J., Park S.-J., Shin D.-H., Kwon C.-H., Shannon J.G., LEE J.D. (2016). Determination of forage quality by near-infrared reflectance spectroscopy in soybean. Turkish J. Agric. For..

[9] Nadeem M., Pham T.H., Nieuwenhuis A., Ali W., Zaeem M., Ashiq W., Gillani S.S.M., Manful C., Adigun O.A., Galagedara L. (2019). Adaptation strategies of forage soybeans cultivated on acidic soils under cool climate to produce high quality forage. Plant Sci..

[10] Hintz R.W., Albrecht K.A., Oplinger E.S. (1992). Yield and quality of soybean forage as affected by cultivar and management practices. Agron. J..

[11] Miller M.D., Edwards R.T., Williams W.A. (1973). Soybeans for Forage and Green Manure.

[12] Pinnamaneni S.R., Anapalli S.S., Reddy K.N., Fisher D.K., Ashwell N.E.Q. (2020). Assessing irrigation water use efficiency and economy of twin-row soybean in the Mississippi Delta. Agron. J..

[13] Bellaloui N., Bruns H.A., Abbas H.K., Mengistu A., Fisher D.K., Reddy K.N. (2015). Effects of row-type, row-spacing, seeding rate, soil-type, and cultivar differences on soybean seed nutrition under US Mississippi delta conditions. PLoS ONE.

[14] Hintz R.W., Albrecht K.A. (1994). Dry matter partitioning and forage nutritive value of soybean plant components. Agron. J..

[15] Munoz A.E., Holt E.C., Weaver R.W. (1983). Yield and quality of soybean hay as influenced by stage of growth and plant density 1. Agron. J..

[16] Buxton D.R., Fales S.L., Fahey G.C. (1994). Plant Environment and Quality. Forage Quality, Evaluation, and Utilization.

[17] Halim R.A., Buxton D.R., Hattendorf M.J., Carlson R.E. (1989). Water-stress effects on alfalfa forage quality after adjustment for maturity differences. Agron. J..

[18] Ohlsson C. (1991). Growth, Development, and Composition of Temperate Forage Legumes and Grasses in Varying Environments, Digital Repository@ Iowa State University. http://lib.dr.iastate.edu/.

[19] Wilson J.R., Minson D.J. (1983). Influence of temperature on the digestibility of the tropical legume Macroptilium atropurpureum. Grass Forage Sci..

[20] Kulkarni K.P., Tayade R., Asekova S., Song J.T., Shannon J.G., Lee J.-D. (2018). Harnessing the potential of forage legumes, alfalfa, soybean, and cowpea for sustainable agriculture and global food security. Front. Plant Sci..

[21] (1989). National Research Council Recommended Dietary Allowances.

[22] Heitholt J.J., Kee D., Farr J.B., Read J.C., Metz S., MacKown C.T. (2004). Forage from soybean provides an alternative to its poor grain yield in the southern Great Plains. Crop. Manag..

[23] Deshavath N.N., Mohan M., Veeranki V.D., Goud V.V., Pinnamaneni S.R., Benarjee T. (2017). Dilute acid pretreatment of sorghum biomass to maximize the hemicellulose hydrolysis with minimized levels of fermentative inhibitors for bioethanol production. 3 Biotech.

[24] Desclaux D., Roumet P. (1996). Impact of drought stress on the phenology of two soybean (*Glycine max* L. Merr) cultivars. Field Crop. Res..

[25] Schenk U., Jäger H., Weigel H. (1997). The response of perennial ryegrass/white clover mini-swards to elevated atmospheric CO_2_ concentrations: Effects on yield and fodder quality. Grass Forage Sci..

[26] Weiss W.P. (1998). Estimating the available energy content of feeds for dairy cattle. J. Dairy Sci..

[27] (2001). National Research Council Nutrient Requirements of Dairy Cattle.

[28] Kuehn C.S., Jung H.G., Linn J.G., Martin N.P. (1999). Characteristics of Alfalfa Hay Quality Grades Based on the Relative Feed Value Index. J. Prod. Agric..

